# Incidence of Metabolic Syndrome over 9 Years Follow-Up; the Importance of Sex Differences in the Role of Insulin Resistance and Other Risk Factors

**DOI:** 10.1371/journal.pone.0076304

**Published:** 2013-09-27

**Authors:** Farzad Hadaegh, Mitra Hasheminia, Mojtaba Lotfaliany, Reza Mohebi, Fereidoun Azizi, Maryam Tohidi

**Affiliations:** 1 Prevention of Metabolic Disorders Research Center, Research Institute for Endocrine Sciences, Shahid Beheshti University of Medical Sciences, Tehran, Iran; 2 Endocrine Research Center, Research Institute for Endocrine Sciences, Shahid Beheshti University of Medical Sciences, Tehran, Iran; University of Louisville, United States of America

## Abstract

To determine, the predictors of incident metabolic syndrome (MetS) in a community-based cohort of West Asians, during a mean follow-up of 9.3 years, a sample of 2858 non-MetS Iranian adults aged ≥ 20 years were examined at baseline and followed at three year intervals during three consecutive phases. The MetS was defined using the joint interim statement. Cox proportional hazard regression was used to determine the independent variables associated with incident MetS. Overall, 1117 new cases MetS were identified resulting in an incidence rate of 550.9/10000 person years (95% CI: 519.5-584.2). The corresponding incidence rates among women and men were 433.5/10000 person years (95% CI: 398.8-471.2) and 749.2/10000 person years (95% CI: 689.9-813.5), respectively. Baseline-adjusted predictors of developing MetS included all of the MetS components, being overweight or obese in both gender, and family history of diabetes and age only in women. There were significant effect modifications of gender on age (P<0.001), high blood pressure (0.026), high waist circumference (P<0001) and obesity categories (all P ≤ 0.01) in multivariate analysis. After considering HOMA–IR in the model, among women, all of the MetS predictors as well as those with HOMA-IR ≥ 2.23 showed a significant risk for incident MetS [HR: 1.63 (1.16-2.28)]; however, among men all the MetS components (WC was marginally significant) as well as the fourth quartile of HOMA-IR [HR: 1.50 (1.03-2.17)] and being overweight showed a significant risk. Finally, in the pooled analysis, we showed that female gender had lower risk for incident MetS than male [HR: 0.58 (0.47-0.70)]. In the Iranian population, high incidence of MetS, especially among men, was shown. Our findings confirmed that sex- specific risk predictors should be considered in primary prevention for incident MetS.

## Introduction

The concept of metabolic syndrome (MetS) as defined by a cluster of risk factors including dysglycemia, central obesity, hypertension and dyslipidemia, is useful in predicting those at risk for cardiovascular disease and diabetes [1-3].

Several cohort studies have been conducted in United States, Europe and East Asia to determine the incidence of MetS and its possible risk factors [4-7]. Data reported by different studies on the predictive powers of MetS components as well as obesity and baseline insulin for incident MetS are not consistent [4,6]. However, no report about the long term incidence of MetS has yet been published from West Asian countries with rapid economic and nutritional transitions leading to high prevalence of risk factors for MetS including obesity [8]. Importantly, more than 30% of The Iranian population suffers from MetS, the prevalence of which is significantly higher among women than in men [9]. Furthermore, we reported 20.4% age-adjusted incident MetS (18.4% male vs. 23.1% women), according to the Adult Treatment Panel III (ATPIII) during approximately 3 years follow-up [10].

Recently, some studies have found sex differences in risk predictors of MetS, suggesting that sex hormone levels and androgen/estrogen balance may play an important role in determining MetS [11,12]. In this study, we aimed to investigate the incidence of MetS and its associated risk factors separately among men and women of The Tehran Lipid and Glucose Study (TLGS) participants during more than 9 years follow-up. Furthermore, we examined whether basal insulin and insulin resistance are important risk factors of incident MetS in each gender.

## Materials and Methods

### Study population

**Figure 1:**
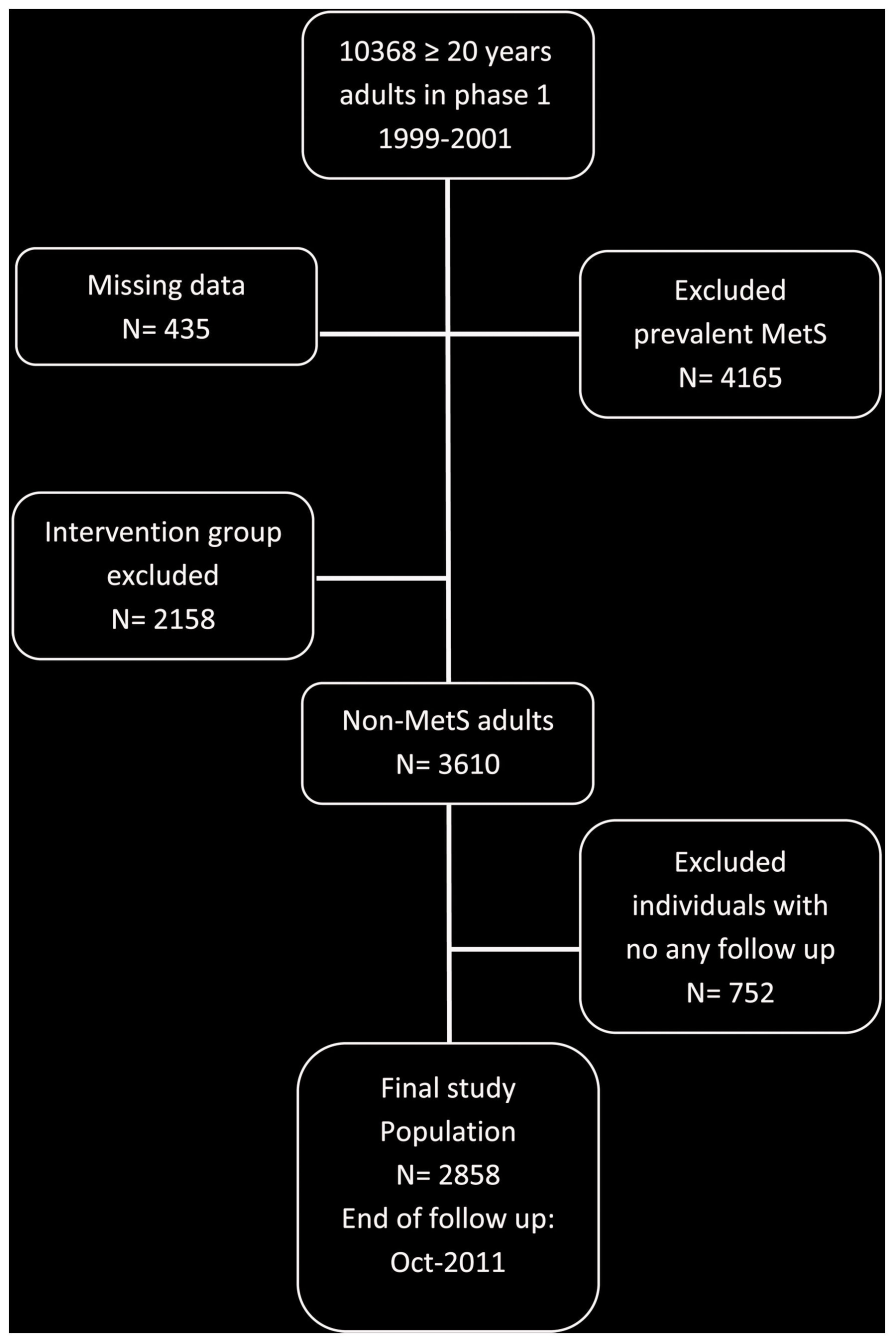
Follow up status of the TLGS participants after the baseline examination. MetS: Metabolic syndrome, TLGS: Tehran Lipid and Glucose Study.

In brief, the TLGS is a large scale, long term, community-based prospective study performed on a representative sample of residents of district No. 13 of Tehran, the capital of Iran [13]. Age and sex distributions of the population in the district were representative of the overall population of Tehran at the time of the baseline examination. The TLGS, has two major components; a cross-sectional prevalence study of non-communicable disease and associated risk factors, initiated between March 1999 and December 2001 (phase 1), and a prospective ongoing follow-up study of participants at three year intervals during three consecutive phases (i.e. phase 2: 2002-2005 ,phase 3: 2005-2008, phase 4: 2008-2011). A total of 10368 residents, aged ≥20 years participated in first examination phase. After exclusion of those with missing data (n=435), prevalent MetS at baseline (n=4165) and those who participated in the intervention group (n=2158), there were 3610 non-MetS adults in the cohort group. Finally, after excluding those lost to follow-up (n=752), 2858 participants were entered in the study (Figure 1). Among this population insulin was measured for 1611 participants (men: 588, women: 1023). The main reasons for lack of attendance at follow-up examinations, despite repeated calls, were either personal or migration. The proposal of this study was approved by the research council of the Research Institute for Endocrine Sciences (RIES) of Shahid Beheshti University of Medical Sciences and written informed consent was obtained from each subject.

### Clinical, anthropometric, and laboratory measurements

Subjects were interviewed privately, by trained interviewers, using pretested questionnaires. Initially, information on demographics, education, smoking status, medical and drug history was collected. Anthropometric measures including weight, height, waist circumference (WC) were obtained, according to standard protocols [13]. Body mass index (BMI) was calculated as weight in kg divided by height squared in m^2^. Systolic and diastolic blood pressures were measured twice in a seated position on the right arm and the mean value was considered as the subject’s blood pressure. A blood sample was taken after 12–14 h overnight fasting and was centrifuged within 30–45 min of collection. All blood analyses were performed at the TLGS research laboratory on the day of blood collection.

Fasting plasma glucose (FPG) was measured by the enzymatic colorimetric glucose oxidase method; inter- and intra-assay coefficients of variation (CV) at baseline and follow-up phases were both less than 2.3%. Total cholesterol (TC) and triglycerides (TG) were assayed using the enzymatic calorimetric method with cholesterol esterase-cholesterol oxidase and glycerol phosphate oxidase, respectively. For TC and HDL- C, intra- and inter-assay CVs were less than 1.9% and 3.0%, respectively in all phases. Intra- and inter-assay CVs were less than 2.1% for TGs at baseline and follow-up examinations. Serum creatinine (Cr) levels were assayed by kinetic colorimetric Jaffe. Intra-assay and inter-assay CVs were both less than 3.1% in both the baseline and follow-up phases. All biochemical assays were performed using commercial kits (Pars Azmoon Inc., Tehran, Iran) by a Selectra 2 auto analyzer (Vital Scientific, Spankeren, The Netherlands). Assay performance was monitored after every 25 tests using lyophilized serum controls in normal and pathologic ranges and all samples were analyzed only when internal quality control met the standard acceptable criteria [13,14]. Fasting serum insulin was determined by the electrochemiluminescence immunoasaay (ECLIA) method using Roche Diagnostics kits & Roche/Hitachi Cobas e- 411 analyzer (GmbH, Mannheim, Germany) with both intra- and inter-assay CVs of less than 3.2%.

### Definition of variables and outcomes

The MetS was defined according to the 2009 scientific consensus, as three or more of the following criteria; TG≥ 150 mg/dl or specific treatment, HDL ≤ 40 mg/dl in men and ≤ 50mg/dl in women or specific treatment, systolic blood pressure ≥130 mmHg or diastolic blood pressure ≥ 85 mmHg or specific treatment, fasting plasma glucose ≥ 100 mg/dl or treatment and high WC using WC cut-off points of ≥90 cm in both genders at risk for CVD risk factors requiring life style change [15,16]. Baseline insulin level (µU/mL) was divided into quartiles in men (insulin< 4.2, 4.2 ≤ insulin <5.8, 5.8≤ insulin <7.7, and insulin ≥7.7) and women (insulin< 5.4, 5.4≤ insulin <7.6, 7.6≤ insulin < 10.2, and insulin ≥ 10.2) and whole population (insulin < 4.9, 4.9≤ insulin <6.8, 6.8≤ insulin<9.4, and insulin ≥9.4) and modeled as a categorical variable, considering the lowest quartile as reference. Insulin resistance (IR) was also estimated by the homeostasis model assessment (HOMA) as an imperfect reference standard for measurement of IR according to the formula [17]: HOMA-IR= [(Fasting insulin level (µU/mL) × FPG (mmol/L)]/ 22.5. HOMA-IR was divided into quartiles in men (HOMA-IR <0.9, 0.9 ≤ HOMA-IR <1.3, 1.3 ≤ HOMA-IR <1.7, and HOMA-IR ≥1.7) and women (HOMA-IR < 1.1, 1.1≤HOMA-IR< 1.6, 1.6 ≤ HOMA-IR < 2.2, and HOMA-IR ≥ 2.2) and in the whole population (HOMA-IR <1.0, 1.0 ≤ HOMA-IR< 1.5, 1.5 ≤ HOMA-IR <2.0, and HOMA-IR ≥ 2.0) and modeled as a categorical variable, considering the lowest quartile as reference.

For this study estimated GFR (eGFR) was calculated using the abbreviated prediction equation, provided by the Modification of Diet in Renal Disease (MDRD) study [18]. Body mass index was categorized into 3 groups of <25 kg/m^2^ (reference), 25 to <30 kg/m^2^ (overweight), and ≥ 30 kg/m^2^ (obese). Smoking was defined in 2 groups; 1.Participants who smoked cigarettes daily or occasionally as well as those who used water pipe or pipe, current or past, as smokers; 2.Those who never smoked (reference). Education was categorized into 3 groups: 1. Illiterate/primary school; 2. Below diploma/diploma and 3. Higher than diploma (reference). Marital status was categorized as single, married (reference) and widowed/divorced. Positive family history of diabetes was defined as having at least one parent or sibling with diabetes. History of cardiovascular disease (CVD) was defined as previous ischemic heart disease and/or cerebrovascular accidents. At baseline examination, the participants were divided into three physical activity groups on the basis of the standards of the Lipid Research Clinic questionnaire. Those who engaged in sports or heavy physical activity at least three times, more than once but less than three times, and less than once per week were defined as having high, moderate, and low physical activity, respectively [19].

### Statistical Analyses

Mean (standard deviation: SD) values for continuous and frequencies (%) for categorical variables of the baseline characteristics are given for participants with and without MetS. Since insulin, FPG and TG had skewed distribution they are shown as median (interquartile range). Comparison of baseline characteristics between participants vs. non-participants, men vs. women and between participants with and without incident MetS were done using student’s t-test for continuous variables, Chi-square test for categorical variables and Mann-Whitney test for skewed variables.

To reduce selection bias [20], propensity scores, estimated probability that a participant would have been followed in the study, were computed using maximum likelihood logistic regression analysis in both genders. For this reason, all baseline measures, including FPG, TG, HDL-C, SBP, DBP, BMI, marital status, education level, history of CVD, family history of diabetes, age, eGFR, WC, drug consumption for diabetes, lipid and hypertension, level of physical activity, smoking and sex (only for the pooled model) were included in a logistic model as exposures with participation in the follow-up as the outcome; the probability of participation in follow-up was then estimated for every participant, each gender and the whole population.

Incidence rates and respective 95% confidence interval (CI) were calculated by dividing the number of events by person-years at risk, for each gender and the whole population. The association of different categorical risk factors with incident MetS was assessed by calculating multivariate adjusted hazard ratios (HRs) with 95% CI using Cox proportional hazard regression analysis. End points were considered as the date of incident MetS and censoring was defined as leaving the residence area, death, lost to follow-up or end of follow up. The event date for the incident cases of MetS was defined as mid-time between the date of follow-up visit at which the MetS was diagnosed for the first time, and the most recent follow-up visit prior to the diagnosis and for those with negative event (censored subjects), the time was the interval between the first and the last observation dates.

For risk factors with more than 2 categories the first category was considered as the reference group. Each candidate predictor (age categories, eGFR categories, marital status, each MetS components, smoking status, history of CVD, family history of diabetes, educational levels and BMI groups and sex (only for analysis of whole population) with a p-value less than 0.2 in the initial univariable analysis was included in the multivariable analysis. The probability of participation in follow-up was used as a propensity score, which were added to the models as a covariate; this probability was not associated with incident MetS in the multivariate models in each gender. The selection bias, therefore, probably did not affect our estimations. Furthermore to examine the independent role of insulin resistance besides other risk factors for incident MetS, we repeated the multivariate analysis in each gender and the whole population in those with complete data for baseline insulin (or HOMA-IR), considering the lowest quartile of insulin (or HOMA-IR) as reference. The effect modifications of gender on the relation between other covariates and MetS outcome were tested by entering the interaction terms (covariate × gender) in the mode. All P-values were two-tailed. We examined the presence of multi-collinearity by calculating the variation inflation factor (VIF) for the independent variables quantified in the regression models. None of the VIFs for the multivariate models exceeded 5, therefore concluding multi-collinearity is unlikely. P values <0.05 were considered statistically significant. Statistical analyses were performed using SPSS program (SPSS Inc., Chicago, IL, USA; Version 15).

## Results

Table 1 shows comparison of baseline characteristics between the followed up versus non- follow up participants as well as men versus women participants. Women participants had higher BMI (25.7 vs. 25.2 kg/m^2^) and lower history of past or current smoking (5.8 vs. 8.7%) compared with nonparticipants. Men participants had lower history of cardiovascular disease (3.2 vs. 5.9%) than nonparticipants. Furthermore, in both genders there was a significant difference in marital status between participants vs. nonparticipants. Comparison between men and women showed that men had higher age, Cr, eGFR, FPG, TG, SBP, WC, smoking rate, family history of cardiovascular disease and lower BMI, HDL-C and baseline insulin level than women. Moreover, there is significant difference between the two genders considering education, physical activity and marital status.

**Table 1:** Comparison of baseline characteristics between the followed up versus non-followed up participants and men versus women participants in the TLGS cohort.

	Men			Women			
	Non-followed up (N=323)	**Followed up (N=1161)**	P-V	Non-Followed up (N=429)	**Followed up (N=1697)**	P-V	**P-V**
Age(years)	40.0 (16.9)	**40.6 (14.9)**	0.478	35.6 (14.1)	**36.1 (12.1)**	0.454	**<0.001**
eGFR(ml/min/1.73m^2^)	75.1 (12.4)	**75.2 (11.6)**	0.903	72.7 (11.9)	**72.2 (11.3)**	0.402	**<0.001**
Cr(mg/dl)	1.2 (0.2)	**1.2 (0.1)**	0.156	1.0 (0.1)	**1.0 (0.1)**	0.943	**<0.001**
FPG(mg)/dl	88.0 (83.0-95.0)	**89.0 (84.0-94.0)**	0.547	86.0 (81.0-91.0)	**87.0 (81.0-92.0)**	0.587	**<0.001**
TG(mg)/dl	115.0 (87.0-144.0)	**118.0 (86.0-155.0)**	0.351	101.0 (77.0-134.0)	**103.0 (77.0-135.0)**	0.645	**<0.001**
HDL_C(mg)/dl	41.7 (11.0)	**41.6 (9.6)**	0.801	47.4 (11.7)	**47.5 (11.4)**	0.862	**<0.001**
SBP(mmHg)	115.4 (16.7)	**114.6 (15.0)**	0.386	111.7 (14.4)	**110.7 (13.2)**	0.173	**<0.001**
DBP(mmHg)	74.2 (11.2)	**74.1 (9.2)**	0.835	74.5 (8.7)	**74.0 (8.8)**	0.365	**0.779**
Waist(cm)	82.3 (10.2)	**82.7 (9.5)**	0.470	80.5 (10.6)	**81.4 (10.7)**	0.116	**0.001**
BMI(kg/m^2^)	23.8 (3.8)	**23.9 (3.5)**	0.876	25.2 (4.5)	**25.7 (4.4)**	0.049	**<0.001**
DM Drug (%)	2.8	**1.1**	0.037	0.9	**0.4**	0.248	**0.037**
Lipid Drug (%)	0.0	**0.2**	1.00	0.7	**0.4**	0.432	**0.326**
HTN Drug (%)	2.2	**2.2**	1.00	2.6	**2.2**	0.718	**0.898**
Marital status (%)			0.025			< .001	**< 0.001**
Married	69.7	**74.2**		68.3	**79.9**		
Divorced /Widowed	1.9	**0.5**		7.5	**4.7**		
Single	28.5	**25.2**		24.2	**15.4**		
HCVD (%)	5.9	**3.2**	0.032	1.4	**1.4**	1.00	**0.001**
FHDM (%)	22.7	**22.1**	0.820	24.1	**25.3**	0.662	**0.054**
Smoking (%)			0.372			0.034	**< 0.001**
Never	54.5	**57.5**		91.3	**94.2**		
Current/Past	45.5	**42.5**		8.7	**5.8**		
Education Level (%)			0.331			0.393	**< 0.001**
Higher than diploma	20.4	**18.4**		13.6	**11.9**		
Diploma/below diploma	60.4	**58.8**		59.7	**63.1**		
Illiterate/Primary School	19.2	**22.8**		26.7	**25.0**		
Physical activity (%)			0.624			0.489	**< 0.001**
Heavy	21.3	**21.7**		29.3	**29.0**		
Light	15.0	**17.1**		11.0	**13.1**		
Never	63.8	**61.2**		59.7	**57.8**		
Insulin (µU/mL)*		**5.8 (4.2-7.7)**			**7.6 (5.4-10.2)**		**<0.001**

P-V: p-value; eGFR: estimated glomerular filtration rate; FPG: fasting plasma glucose; TG: triglyceride; HDL-C: High density lipoprotein; SBP: systolic blood pressure; DBP: diastolic blood pressure; BMI: body mass index; DM: diabetes mellitus; HTN: hypertension; HCVD: history of cardiovascular disease; FHDM: family history of diabetes mellitus. Cr: serum creatinine

* Insulin was measured in 1611 participants (men: 588, women: 1023)

The comparison between men versus women participants was been bolded.

Table 2 shows the comparison of baseline characteristics of participants, with and without incident MetS. In both genders, the participants who developed incident MetS were older, had higher SBP and DBP, TG, FPG, basal insulin, BMI, WC and positive family history of diabetes but lower eGFR and HDL-C compared with participants free of MetS at the end of follow-up (P<0.05 for all measures). Furthermore, women with incident MetS showed higher serum Cr and positive family history of cardiovascular disease than the non-MetS group.

**Table 2:** Comparison of baseline characteristics of subjects who did and did not develop incident MetS after 9.3 years of follow-up.

	Men			Women		
	Non-MetS (N=596)	MetS (N=565)	P-V	Non-MetS (N=1145)	MetS (N=552)	P-V
Age(years)	39.5 (15.6)	41.9 (14.0)	0.007	33.3 (10.9)	41.9 (12.3)	<0.001
eGFR(ml/min/1.73m^2^)	76.0 (11.9)	74.3 (11.3)	0.016	73.9 (11.0)	68.7 (11.0)	<0.001
Cr(mg/dl)	1.2 (0.1)	1.2 (0.1)	0.441	1.0 (0.1)	1.0 (0.1)	0.001
FPG(mg/dl)	88.0 (83.0-92.8)	90.0 (85.0-96.0)	<0.001	85.0 (80.0-90.0)	89.0 (84.0-94.0)	<0.001
TG(mg/dl)	99.0 (75.0-134.0)	135.0 (106.5-176.5)	<0.001	93.0 (70.0-121.0)	127.5 (100.3-159.8)	<0.001
HDL_C(mg/dl)	43.6 (9.8)	39.4 (9.0)	<0.001	48.7 (11.3)	45.2 (11.2)	<0.001
SBP(mmHg)	112.8 (14.9)	116.5 (14.8)	<0.001	108.6 (11.9)	115.2 (14.7)	<0.001
DBP(mmHg)	72.8 (9.4)	75.5 (8.8)	<0.001	72.8 (8.5)	76.6 (9.0)	<0.001
Waist(cm)	79.9 (9.7)	85.6 (8.3)	<0.001	78.4 (10.0)	87.5 (9.4)	<0.001
BMI(kg/m^2^)	22.9 (3.5)	24.8 (3.1)	<0.001	24.6 (4.1)	27.9 (4.0)	<0.001
DM Drug (%)	0.3	1.9	0.011	0.1	1.1	0.006
Lipid Drug (%)	0.0	0.4	0.237	0.1	1.1	0.006
HTN Drug (%)	1.3	3.0	0.067	1.4	4.0	0.001
Marital status (%)			< 0.001			< 0.001
Married	68.0	80.9		76.5	87	
Divorced/Widowed	0.3	0.7		3.3	7.4	
Single	31.7	18.4		20.2	5.6	
HCVD (%)	2.5	3.9	0.186	0.9	2.4	0.023
FHDM (%)	18.3	26.2	0.001	22.4	31.2	< 0.001
Smoking (%)			0.720			0.656
Never	58.0	56.9		94.4	93.8	
Current/Past	42.0	43.1		5.6	6.2	
Education Level (%)			0.256			< 0.001
Higher than diploma	19.3	17.3		13.5	8.3	
Diploma/below diploma	56.5	61.2		68.3	52.5	
Illiterate/Primary School	24.2	21.4		18.2	39.2	
Physical activity (%)			0.797			0.802
Heavy	21.5	21.9		28.7	29.6	
Light	16.4	17.8		12.9	13.6	
Never	62.0	60.3		58.4	56.7	
Insulin (µU/mL)*	5.3 (3.6-7.5)	6.1 (4.6-8.4)	0.001	7.2 (5.1-9.6)	8.1 (5.9-11.4)	<0.001

P-V: p-value; eGFR: estimated glomerular filtration rate; FPG: fasting plasma glucose; TG: triglyceride; HDL-C: High density lipoprotein; SBP: systolic blood pressure; DBP: diastolic blood pressure; BMI: body mass index; DM: diabetes mellitus; HTN: hypertension; HCVD: history of cardiovascular disease; FHDM: family history of diabetes mellitus; Cr: serum creatinine

* Insulin was measured in 1611 participants (men: 588, women: 1023)

Overall, 1117 new cases MetS (women: 552, men: 565) were identified after a mean follow-up of 9.3 years resulting in an incidence rate of 550.9/10000 person years (95% CI: 519.5-584.2). The incidence rate of MetS among women [433.5/10000 person years (95% CI: 398.8-471.2)] was significantly lower than men population [749.2/10000, (95% CI: 689.9-813.5)], (P<0001). There were significant effect modifications of gender on age (P<0.001), High BP (0.026), High WC (P<0001) and obesity categories (P ≤ 0.01) in multivariate analysis. Hence, we stratified our analysis by gender. Insulin level and HOMA-IR, however, did not show significant interactions with sex when examined as a categorical variable. Also, for our findings to be comparable to other studies, we showed our data analyses in the whole population as well.

Table 3 shows the adjusted HRs of MetS associated with baseline risk factors in each gender and the whole population in models with and without HOMA-IR. All Mets components, being overweight or obese and positive history of diabetes mellitus (marginally significant in men) were significant predictors of developing MetS in each gender and pooled models. Furthermore, age was an independent predictor only in women [HRs: 1.02 (1.01-1.03)] and pooled [HR: 1.01 (1.01-1.02)] models. Moreover in the pooled model, female gender had lower risk for incident MetS than male [HRs: 0.54 (0.47-0.63)]. After considering HOMA –IR in the model, among women, all of the MetS predictors as well as those with HOMA-IR ≥ 2.2 showed a significant risk for incident MetS [HR: 1.63 (1.16-2.28)]; however, among men all the MetS components (WC was marginally significant, [HR: 1.47 (0.97-2.22)]) as well as the fourth quartile of HOMA-IR [HR: 1.50 (1.03-2.17)] and being overweight showed a significant risk. Importantly in the pooled sample, 2^nd^, 3^th^ and 4^th^ categories of HOMA-IR as well as all Mets components, positive family history of diabetes and being overweight/obese, age and male gender all highlighted significant risk for incident MetS. Similarly, when we entered baseline insulin in place of HOMA-IR in the multivariable model, the second and fourth quartiles of baseline insulin remained as significant predictors in women, while among men, only the second quartile of baseline insulin increased the risk of incident MetS. On the other hand in the pooled model, the second and fourth quartiles of baseline insulin remained as significant predictors in participants (Table S1).

**Table 3:** The predictors for developing MetS in each gender and the whole population in models with and without HOMA-IR.

	Men		Women		Whole population	
	Hazard ratio (CI)	Hazard ratio (CI)	Hazard ratio (CI)	Hazard ratio (CI)	Hazard ratio (CI)	Hazard ratio (CI)
Age(years)	1.01 (0.99-1.01)	1.01 (0.99-1.02)	1.02 (1.01-1.03)	1.03 (1.02-1.04)	1.01 (1.01-1.02)	1.02 (1.01-1.03)
Gender						
Male					Reference	Reference
Female					0.54 (0.47-0.63)	0.58 (0.47-0.70)
eGFR(ml/min/1.73m^2^)						
< 60 (1)	0.95 (0.61-1.47)	1.10 (0.61-1.98)	1.25 (0.73-2.12)	0.77 (0.41-1.44)	1.12 (0.81-1.53)	0.96 (0.64-1.44)
≥ 60 &<75 (2)	0.91 (0.66-1.25)	0.96 (0.63-1.46)	0.11 (0.69-1.80)	0.89 (0.51-1.55)	0.95 (0.73-1.23)	0.92 (0.66-1.28)
≥75 &<90 (3)	0.88 (0.64-1.20)	0.94 (0.62-1.41)	0.99 (0.61-1.61)	0.75 (0.43-1.31)	0.88 (0.68-1.14)	0.83 (0.59-1.15)
≥ 90 (4)	Reference	Reference	Reference	Reference	Reference	Reference
Marital status						
Married	Reference	Reference	Reference	Reference	Reference	Reference
Divorced / Widowed	1.58 (0.58-4.29)	2.04 (0.62-6.75)	0.73 (0.51-1.04)	0.68 (0.41-1.12)	1.06 (0.77-1.46)	1.02 (0.66-1.58)
Single (2)	0.82 (0.62-1.07)	0.81 (0.56-1.17)	0.72 (0.49-1.07)	0.92 (0.58-1.45)	0.89 (0.72-1.10)	1.02 (0.78-1.34)
HCVD						
No	Not Applicable*	Not Applicable*	Reference	Reference	Reference	Reference
Yes			0.96 (0.57-1.82)	1.01 (0.49-2.05)	1.026	0.80 (0.49-1.30)
Education Level						
Higher than diploma	Reference	Reference	Reference	Reference	Reference	Reference
Diploma/below diploma (2)	0.99 (0.74-1.32)	0.82 (0.54-1.24)	1.38 (0.97-1.96)	1.32 (0.84-2.08)	1.24 (1.00-1.54)	1.18 (0.89-1.57)
Illiterate/Primary School(1)	1.20 (0.95-1.51)	1.19 (0.87-1.63)	1.18 (0.86-1.63)	1.07 (0.71-1.60)	1.18 (0.98-1.42)	1.12 (0.88-1.43)
High TG						
No	Reference	Reference	Reference	Reference	Reference	Reference
Yes	2.05 (1.70-2.48)	1.89 (1.45-2.46)	2.81 (2.27-3.47)	2.87 (2.19-3.78)	2.45 (2.13-2.83)	2.52 (2.09-3.03)
High FPG						
No	Reference	Reference	Reference	Reference	Reference	Reference
Yes	2.03 (1.53-2.69)	1.84 (1.21-2.77)	2.70 (1.94-3.75)	2.38 (1.50-3.76)	2.23 (1.80-2.76)	2.11 (1.56-2.86)
High Waist						
No	Reference	Reference	Reference	Reference	Reference	Reference
Yes	1.41 (1.07-1.87)	1.47 (0.97-2.22)	2.21 (1.72-2.84)	2.36 (1.72-3.25)	1.76 (1.46-2.11)	2.73 (1.35-2.22)
High BP						
No	Reference	Reference	Reference	Reference	Reference	Reference
Yes	1.71 (1.34-2.18)	1.48 (1.03-2.12)	2.07 (1.60-2.70)	2.08 (1.49-2.91)	1.85 (1.55-2.21)	2.69 (1.33-2.16)
Low HDL_C						
No	Reference	Reference	Reference	Reference	Reference	Reference
Yes	1.79 (1.48-2.15)	1.62 (1.25-2.11)	1.99 (1.61-2.47)	2.16 (1.64-2.85)	1.86 (1.61-2.14)	2.81 (1.50-2.19)
BMI(kg/m^2^)						
< 25	Reference	Reference	Reference	Reference	Reference	Reference
≥25 &< 30 (1)	1.74 (1.41-2.15)	1.61 (1.19-2.18)	1.98 (1.59-2.48)	2.09 (1.57-2.78)	1.84 (1.59-2.13)	1.83 (1.51-2.23)
≥ 30 (2)	1.67 (1.07-2.60)	1.09 (0.56-2.13)	2.18 (1.62-2.92)	2.23 (1.53-3.25)	2.10 (1.66-2.65)	1.99 (1.46-2.70)
Smoking						
Never	Reference	Reference	Not Applicable*	Not Applicable*	Reference	
Past / Current	0.97 (0.81-1.15)	1.02 (0.80-1.32)			0.96 (0.82-1.12)	1.02 (0.82-1.26)
FHDM						
No	Reference	Reference	Reference	Reference	Reference	Reference
Yes	1.20 (0.99-1.15)	1.14 (0.87-1.49)	1.29 (1.08-1.56)	1.53 (1.22-1.92)	1.26 (1.11-1.44)	1.37 (1.16-1.63)
HOMA-IR**						
HOMA-IR group		Reference		Reference		Reference
HOMA-IR group (1)		1.25 (0.86-1.79)		1.38 (0.98-1.94)		1.56 (1.22-1.99)
HOMA-IR group (2)		1.38 (0.95-2.00)		1.36 (0.96-1.92)		1.36 (1.02-1.72)
HOMA-IR group (3)		1.50 (1.03-2.17)		1.63 (1.16-2.28)		1.57 (1.22-2.04)

eGFR: estimated glomerular filtration rate; FPG: fasting plasma glucose; TG: triglyceride; HDL-C: High density lipoprotein, BP: blood pressure; BMI: body mass index; DM: diabetes mellitus; HTN: hypertension; HCVD: history of cardiovascular disease; FHDM: family history of diabetes mellitus; HOMA-IR: Insulin resistance estimated by the homeostasis model assessment

* P value > 0.2 in univariable analysis;

** Insulin was measured in 1611 participants (men: 588, women: 1023)

## Discussion

In this cohort, the incidence of Mets was 550.9/10,000 person -year (95% CI: 519.5-584.2), which was significantly higher among men than women (749.2/10000 vs. 433.5/10000 person years). Among both genders, all MetS components, family history of diabetes and being overweight or obese were independent predictors for incident MetS, however, the effect of age, high BP, high WC and the obesity categories were greater among women than in men. Importantly, women showed about 50% lower risk for MetS than men. Furthermore, the fourth quartile of HOMA-IR was associated with significant risk in both genders.

Data reveals that high incidence of MetS among Iranian populations could be due to life style changes including dietary habits and physical activity patterns, that have been occurring very often in recent years due to rapid urbanization and Westernization of life style, leading to a sharp rise in risk factors of chronic disease [21]. In Iran, carbohydrates, particularly in the form of refined grains, which usually have higher amounts of glycemic index and glycemic load, are the main dietary component; inappropriate carbohydrate intake and quality might contribute to increased levels of serum triglycerides and decreased levels of serum HDL-C, leading to high incidence of MetS [22]. MetS is an independent predictor for incident diabetes and CVD among Iranian populations; the mortality rate attributable to these diseases has been estimated to be more than 400 per 100,000 in Iran [1,2,23]. To our knowledge, limited research has been done to estimate the incidence of MetS using the JIS definition, in other populations, which is why we cannot compare our results with those of other populations.

The incidence rate of metabolic syndrome applying AHA/NHLBI criteria in an urban South European population was 47.2/1000 person-years, similar in females and in males [24]. Also, during 10 years of follow-up among Japanese–American the cumulative incidence of MetS was 17.8% [25]. We reported earlier that MetS showed higher prevalence among women than in men using International Diabetes Federation (IDF) and National Cholesterol Education Program (NCEP) definitions [9]. In the current study, however, the incidence of MetS was significantly higher among men, which might be attributable to higher baseline levels of WC, FPG, TG, SBP and lower levels of HDL-C among men compared to women. Furthermore, Hoseinpanah et al [26] showed that the trends of obesity and abdominal obesity are more alarming in men than in women, which might be due to the importance given by women to their health, because of both their increasing educational levels and higher income in recent years; additionally, it can also be a result of public educational programs being focused more on women as the target group because of their higher obesity levels, compared to men. This higher trend of general and abdominal adiposity among men than women might be translated to the significant risk of incident MetS among men than women. Limited data is available on the gender differences in MetS incidence, particularly among West Asian populations with a high prevalence of the syndrome. While some studies report higher incidence of MetS in men, others conducted in women found no significant difference between genders [5,7,24,25].

Family history of diabetes among both genders independently increased the risk of MetS by more than 20%. Studies show that MetS has both genetic and environmental bases. In fact in the TLGS population, the highest heritability among MetS components was for HDL-C and TG [27]. Our finding extends our recent findings showing that family history of diabetes might be a surrogate of genetic basis has an independent role for incident MetS.

In this study, we did not find any association between creatinine and eGFR with incident MetS, even in age adjusted analysis. Also, we did not find any risk for eGFR or creatinine level categories in the MetS component adjusted model (Data available on request). In line with our findings, Onat et al [28] showed that MetS was not significantly associated with a reduced eGFR category when controlled for homeostatic model assessment (HOMA) in a cross-section study. Recently some studies have shown that elevated CHD and type 2 diabetes risk were associated with serum creatinine [29,30]. Furthermore, glomerular hyper filtration which was defined as estimated creatinine clearance over the mean + 2SD was related to a high cardiovascular risk profile [31]. With respect to CHD among Turkish women, also, a significant risk residual to MetS was reported [32] ultimately identified as being due to autoimmune activation [33]

Insulin resistance, as described by Raven et al, was reported as an initial cause of MetS [25,34]. In our study fasting insulin was applied as a surrogate marker of insulin resistance leading to different combinations of fasting insulin and the glucose concentration such as HOMA-IR [35-37]. We found that highest quartile of HOMA-IR and baseline insulin had about 50% and 40% increased risk for incident MetS, compared to that of the subjects in the lowest quartile sex –adjusted analysis, respectively. In a study conducted among non-obese, non-diabetic Japanese-Americans, insulin resistance, as measured by either fasting insulin or HOMA-IR and intra-abdominal fat, were both good predictors for the 10 year incidence of MetS in sex adjusted analysis [25].

In this study, the WC as a core component in the IDF definition and general adiposity whether categorized as overweight or obesity were suggested as independent predictors. Similar findings were shown in Korean male workers, South European populations and The Resistance Atherosclerosis Study [4,24,38]. Additionally we showed that the effect of general and central adiposity for incident MetS was significantly higher among women than men.

Regarding social status, there is no significant correlation between incident MetS and marital or educational status. In contrast, some cross-sectional studies conducted in Iranian and other populations showed MetS prevalence to be significantly higher among married compared to unmarried populations [39-41]. Furthermore in South European and the British household panel survey populations less educated participants had a higher syndrome incidence [24,42]. Considering unhealthy behavior, in our data analysis history of smoking (current or past) did not have significant association with incident MetS. Correspondingly, in the Tromso study, smoking as a binary variable was not associated with MetS [5]. On the other hand, in a recent meta-analysis involving 56,691 participants and 8,688 cases, Sun et al found a significant positive association between active smoking and risk of metabolic syndrome, the risk was stronger for active male smokers than it was for former male smokers and greater for heavy smokers, compared to light smokers [43].

Of other components of MetS, low HDL-C, with the highest prevalence in our population, resulted in an over 2 fold increase in risk of MetS in both genders [9]. Importantly, high blood pressure in both genders was a very strong predictor of incident MetS, even in the presence of HOMA-IR or baseline insulin as well as general and central obesity, suggesting that etiology of hypertension among Iranian populations is more closely related to insulin resistance than in some other populations [6,44] and those who are insulin resistant would be expected to have other components of MetS. Moreover, we recently reported that WC is an independent predictor for incident hypertension among Iranian women [45].

We found no association between physical activity and incident MetS during over 9 years follow-up, a finding also reported in another short term study conducted among Korean male workers [38] and in the IRAS [4]. However, the change in life style during follow-up which affects weight gain, as an independent and strong predictor of incidence MetS, was not considered in the current study [10].

The current study has some limitations. We measured baseline characteristics of the participants only once, and hence misclassification of potential risk factors might have attenuated our estimates. Furthermore, there are uncertainties, which might restrict extrapolation of our findings to other geographic regions of the country.

To conclude, during over 9 years of follow-up, the incidence rates of MetS among men and women were 749.2/10000 and 433.5/10000 person years, respectively. In both genders, all MetS components, positive history of diabetes, being overweight or obese and age (among women) were found to be independent predictors of MetS. Moreover, the highest quartile of HOMA-IR and insulin level was an independent predictor of MetS. Our findings confirm that sex-specific risk predictors should be considered in primary prevention for incident MetS among Iranian populations.

## Supporting Information

Table S1 **The predictors for developing MetS in each gender and the whole population in models with baseline serum insulin.**
(DOCX)
