# Supplementary material for: Incidence of Metabolic Syndrome over 9 Years Follow-Up; the Importance of Sex Differences in the Role of Insulin Resistance and Other Risk Factors
Source: PLoS One. 2013 Sep 27;8(9):e76304. doi: 10.1371/journal.pone.0076304 (PMC3785433; doi:10.1371/journal.pone.0076304)
Supplement: Table S1 — The predictors for developing MetS in each gender and the whole population in models with baseline serum insulin. (DOCX) [file pone.0076304.s001.docx]

**Supporting information Table S1:** **The predictors for developing MetS in each gender and the whole population in models with baseline serum insulin.**

|  | Men | Women | Whole population | |
| --- | --- | --- | --- | --- |
|  | Hazard ratio (CI) | Hazard ratio (CI) | Hazard ratio (CI) | |
| Age(years) | 1.01(0.99-1.02) | 1.03(1.02-1.04) | 1.02(1.01-1.03) | |
| Gender |  |  |  | |
| Male |  |  | Reference | |
| Female |  |  | 0.57(0.46-0.69) | |
| eGFR(ml/min/1.73m^2^) |  |  |  | |
| < 60 (1) | 1.11(0.62-1.99) | 0.77(0.41-1.44) | 0.95(0.64-1.43) | |
| ≥ 60 &<75 (2) | 0.94(0.62-1.44) | 0.90(0.52-1.55) | 0.91(0.65-1.26) | |
| ≥75 &<90 (3) | 0.92(0.61-1.39) | 0.76(0.43-1.33) | 0.81(0.58-1.13) | |
| ≥ 90 (4) | Reference | Reference | Reference | |
| Marital status |  |  |  | |
| Married | Reference | Reference | Reference | |
| Divorced / Widowed (1) | 1.92(0.58-6.35) | 0.67(0.41-1.10) | 1.06(0.68-1.65) | |
| Single (2) | 0.81(0.56-1.66) | 0.92(0.58-1.46) | 1.03(0.79-1.35) | |
| HCVD |  |  |  | |
| No | Not Applicable* | Reference | Reference | |
| Yes |  | 1.00(0.49-2.03) | 0.82(0.50-1.33) | |
| Education Level |  |  |  | |
| Higher than diploma | Reference | Reference | Reference | |
| Diploma/ below diploma | 0.83(0.54-1.26) | 1.35(0.86-2.12) | 1.21(0.91-1.62) | |
| Illiterate/ Primary School(1) | 1.19(0.87-1.63) | 1.07(0.71-1.60) | 1.14(0.89-1.45) | |
| High TG |  |  |  | |
| No | Reference | Reference | Reference | |
| Yes | 1.92(1.48-2.50) | 2.94(2.24-3.86) | 2.61(2.17-3.15) | |
| High FPG |  |  |  | |
| No | Reference | Reference | Reference | |
| Yes | 1.98(1.32-2.97) | 2.58(1.64-4.08) | 2.24(1.65-3.02) | |
| High Waist |  |  |  | |
| No | Reference | Reference | Reference | |
| Yes | 1.48(0.98-2.23) | 2.37(1.72-3.26) | 1.78(1.39-2.28) | |
| High BP |  |  |  | |
| No | Reference | Reference | Reference | |
| Yes | 1.48(1.03-2.13) | 2.09(1.50-2.93) | 1.69(1.33-2.16) | |
| Low HDL_C |  |  |  | |
| No | Reference | Reference | Reference | |
| Yes | 1.62(1.25-2.10) | 2.14(1.63-2.83) | 1.83(1.51-2.21) | |
| BMI(kg/m2) |  |  |  | |
| < 25 | Reference | Reference | Reference | |
| ≥25 &< 30 (1) | 1.62(1.19-2.20) | 2.12(1.59-2.82) | 1.89(1.56-2.30) |  |
| ≥ 30 (2) | 1.13(0.58-2.23) | 2.27(1.55-3.30) | 2.05(1.51-2.79) |  |
| Smoking |  |  |  |  |
| Never | Reference | Not Applicable* | Reference­ |  |
| Past / Current | 1.02(0.80-1.31) |  | 1.00(0.81-1.24) |  |
| FHDM |  |  |  |  |
| No | Reference | Reference­ | Reference­ |  |
| Yes | 1.15(0.88-1.50) | 1.51(1.20-1.89) | 1.35(1.14-1.61) |  |
| Insulin |  |  |  |  |
| Insulin group** | Reference | Reference­ | Reference­ |  |
| Insulin group (1) | 1.45(1.02-2.08) | 1.41(1.01-1.96) | 1.35(1.07-1.71) |  |
| Insulin group (2) | 1.38(0.95-2.01) | 1.14(0.81-1.61) | 1.05(0.82-1.35) |  |
| Insulin group (3) | 1.43(0.97-2.09) | 1.49(1.07-2.08) | 1.39(1.08-1.78) |  |

eGFR: estimated glomerular filtration rate; FPG: fasting plasma glucose; TG: triglyceride; HDL-C: High density lipoprotein, BP: blood pressure; BMI: body mass index; DM: diabetes mellitus; HTN: hypertension; HCVD: history of cardiovascular disease; FHDM: family history of diabetes mellitus; HOMA-IR: Insulin resistance estimated by the homeostasis model assessment

* P value > 0.2 in univariable analysis

** Insulin was measured in 1611 participants (men: 588, women: 1023)
